# Exploring oxide quasicrystals in internal space

**DOI:** 10.1107/S2053273325011015

**Published:** 2026-01-09

**Authors:** Sebastian Schenk, Martin Haller, Stefan Förster, Wolf Widdra

**Affiliations:** a Institute of Physics, Martin-Luther-Universität Halle-Wittenberg, D-06099 Halle, Germany; Université de Lorraine, France

**Keywords:** tiling analysis, phason elastic constant, perpendicular space, hyperspace, phason flips, quasicrystals, random tiling, parallel space

## Abstract

Quantitative assessment of the structural quality of quasicrystals is a challenging task. Whereas diffraction techniques are a powerful tool for the coherently scattering regions, only diffuse scattering addresses structural inhomogeneities. Alternatively, local imaging techniques allow for direct inspection of the structures and for statistical evaluation of tiling elements. In both cases, inspecting the internal space characteristic for quasicrystals offers an additional, more sensitive perspective. Here, we analyze atomically resolved scanning tunneling microscopy images for three dodecagonal oxide quasicrystal (OQC) systems. Upon uplifting the 2D coordinates into a 4D hyperspace, the quasicrystal structure can be discussed in the internal space as well as in the physical space. The quasicrystal acceptance domain in internal space of all three OQCs increases logarithmically with the system size. From the acceptance domain expansion we determine the effective phason elastic constants, which reflect phason disorder within their square–triangle–rhombus tiling.

## Abstract 2

The internal space expansion with variable system size is investigated for three different oxide quasicrystal systems. Patches of 7800, 4800 and 3600 vertices are examined in Ba–Ti–O/Pt(111), Eu–Ti–O/Pd(111) and Sr–Ti–O/Pd(111), respectively. This internal space inspection provides unique structural information for quasicrystalline systems, which goes far beyond a tiling analysis in the physical space.

## 1. Introduction

Quasicrystals represent a unique state of matter owing to their unconventional symmetries, allowing *e.g.* five-, eight-, ten- or 12-fold rotational diffraction patterns. Although they are aperiodic in physical space, quasicrystals are periodic in a higher-dimensional space called superspace. As a consequence of this superspace description, all vertices of the high-dimensional lattice can be represented either in the physical space, where they are aperiodically arranged, or in an internal space, which is a space of the same dimensions perpendicular to the physical space. Inspection of the internal space offers a unique tool for the structural analysis of quasicrystals. An ideal quasicrystal appears as a dense set of points in the internal space that defines its window or acceptance domain (AD). Any defects in the quasicrystal will cause points to scatter outside this acceptance domain. In this way, phason disorder, dislocations, phason strain and topological defects can be revealed (Socolar *et al.*, 1986 ▸; He *et al.*, 1991 ▸; Hory *et al.*, 1999 ▸; Edagawa *et al.*, 2000 ▸; Edagawa *et al.*, 2002 ▸; Blunt *et al.*, 2008 ▸; Lifshitz, 2011 ▸; Korkidi *et al.*, 2013 ▸; Sandbrink & Schmiedeberg, 2014 ▸; Ishimasa *et al.*, 2015 ▸; Hielscher *et al.*, 2017 ▸; Hielscher *et al.*, 2020 ▸).

The internal space analysis enables random quasicrystals to be distinguished. To this end, the variance or mean-squared expansion 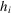 of the internal space distribution is investigated as a function of system size *N* (Henley, 1991 ▸; Goldman & Kelton, 1993 ▸; Joseph & Baake, 1996 ▸). It is defined as 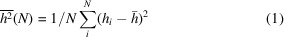
according to Joseph & Baake (1996 ▸). The following cases can be distinguished. The variance 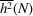 is a constant for a deterministic quasicrystal. It grows in a parabolic fashion in the case of linear phason strain, which describes periodic quasicrystal approximants. For random tiling quasicrystals the variance grows differently depending on the internal space dimensions. In 1D, the problem is equivalent to a random walk, meaning the variance is proportional to the chain length. In 2D, as concluded from the hydrodynamic approximation, the variance should scale as 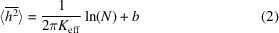
with an effective elastic constant 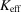, the number of vertices *N* and an integration constant *b*. For 3D a power-law finite-size scaling is expected (Joseph & Baake, 1996 ▸). An analysis of the scaling of the internal space variance has been applied previously to a number of systems, including decagonal Al–Ni–Co and Al–Cu–Ce quasicrystals, dodecagonal molecular self-assemblies and dodecagonal Mn–Cr–Ni–Si (Chen *et al.*, 1990 ▸; He *et al.*, 1991 ▸; Joseph *et al.*, 1997 ▸; Blunt *et al.*, 2008 ▸; Ishimasa *et al.*, 2015 ▸). Most recently, it has been applied to dodecagonal pentablock quarterpolymers (Matsushita *et al.*, 2024 ▸).

A more subtle analysis of the tiling can be derived from the Fourier transform (FT) of the internal space distribution (Joseph & Baake, 1996 ▸). The advantage of the FT is that it uses all the statistical moments of the point cloud in space; the variance 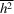, on the other hand, is equivalent to the second moment only. In the case of an ideal quasicrystal, the absolute value of the discrete FT of the internal space point cloud is expected to have a central Gaussian peak surrounded by oscillations. In contrast, there are no oscillations for the random tiling (Joseph & Baake, 1996 ▸).

Here, we present an internal space analysis of three different oxide quasicrystal (OQC) systems. OQCs are 2D ternary oxides on a metal support that exhibit a dodecagonal square–triangle–rhombus tiling (Förster *et al.*, 2013 ▸; Förster *et al.*, 2020 ▸). The most thoroughly investigated OQC system is Ba–Ti–O/Pt(111). In this materials system OQCs have been discovered and their growth has been optimized to the highest level of structural perfection (Zollner *et al.*, 2020 ▸). Furthermore, the physical space of Ba–Ti–O/Pt(111) has previously been analyzed in great detail in terms of tiling statistics and its hyperslope as calculated from the tiling element distribution (Schenk *et al.*, 2019 ▸; Zollner *et al.*, 2020 ▸; Imperor-Clerc *et al.*, 2024 ▸). One important finding is that this square–triangle–rhombus tiling obeys the statistics of the ideal Niizeki–Gähler tiling (NGT) with respect to the allowed vertex configurations and the relative frequencies of the prototiles (Schenk *et al.*, 2019 ▸; Niizeki & Mitani, 1987 ▸; Gähler, 1988 ▸). However, systematic variations in the orientational distribution of larger clusters, namely the characteristic dodecagon consisting of two rhombuses, five squares and 12 triangles, were reported for the Ba–Ti–O/Pt(111) system (Schenk *et al.*, 2019 ▸). In addition to this prototypical OQC system, we perform the internal analysis for Eu and Sr atoms in a quasicrystalline network formed on Pd(111) (Schenk *et al.*, 2022 ▸; Haller *et al.*, 2025 ▸). We observe a significant scattering of points outside the AD for all three dodecagonal structures. From the scaling of the mean-squared expansion when plotted against the number of vertices, we obtain a limited size for the well ordered quasicrystal patches and determine an effective phason elastic constant 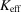.

## 2. Experimental

The Ba–Ti–O/Pt(111) OQC has been prepared by depositing ultrathin BaTiO_3_ films via magnetron sputtering and annealing at 1150 K in ultrahigh vacuum (UHV). The structural aspects as determined by low-energy electron diffraction (LEED) and scanning tunneling microscopy data have been discussed previously (Schenk *et al.*, 2019 ▸). For Sr–Ti–O and Eu–Ti–O OQCs on Pd(111), monolayers of TiO_*x*_ were prepared by molecular beam epitaxy of Ti and post-oxidation. Subsequently, Sr/Eu were deposited to an extent that the entire oxide monolayer transforms into the dodecagonal OQCs (Haller *et al.*, 2025 ▸).

Scanning tunneling microscopy (STM) data were acquired at 77 K. The raw data were corrected for piezo creep and thermal drift and afterwards were processed using *Mathematica* (Schenk *et al.*, 2019 ▸). A custom analysis pipeline was used to determine the Ba, Sr and Eu vertex positions. First, the STM images were binarized and gradients were applied to the resulting vertex discs. Then, a watershed segmentation algorithm partitioned the image into vertex regions. Finally, the vertex coordinates were refined by fitting 2D Gaussian functions to the segmented regions. After extracting the exact positions, the vertices were indexed using a 4D basis. In an ideal quasicrystal, the coordinates of the vertices are restricted to integers, providing a constraint for the indexing procedure. To initiate this process, a list of neighboring vertices was generated and the mean nearest-neighbor distance was computed. This characteristic length was then used to normalize all pairwise distances in the dataset. An arbitrary vertex located near the center of the STM image was selected as the origin and given the index (0, 0, 0, 0). The remaining vertices were ordered according to their distance from the origin; those closer were given higher priority. Indexing was then performed iteratively. Starting with the origin, each vertex was evaluated in turn. If neighboring vertices had not yet been indexed, it was determined whether their positions could be reached by adding a unit vector to the reference vertex’s index. If this condition was satisfied, the neighbor was assigned the corresponding 4D index. This neighbor-based, iterative indexing scheme assigns unique integer coordinates to all vertices using only local geometric information. Since it relies exclusively on local neighborhoods, the method remains robust against global distortions of the STM images.

## 3. Results

**Figure 1:**
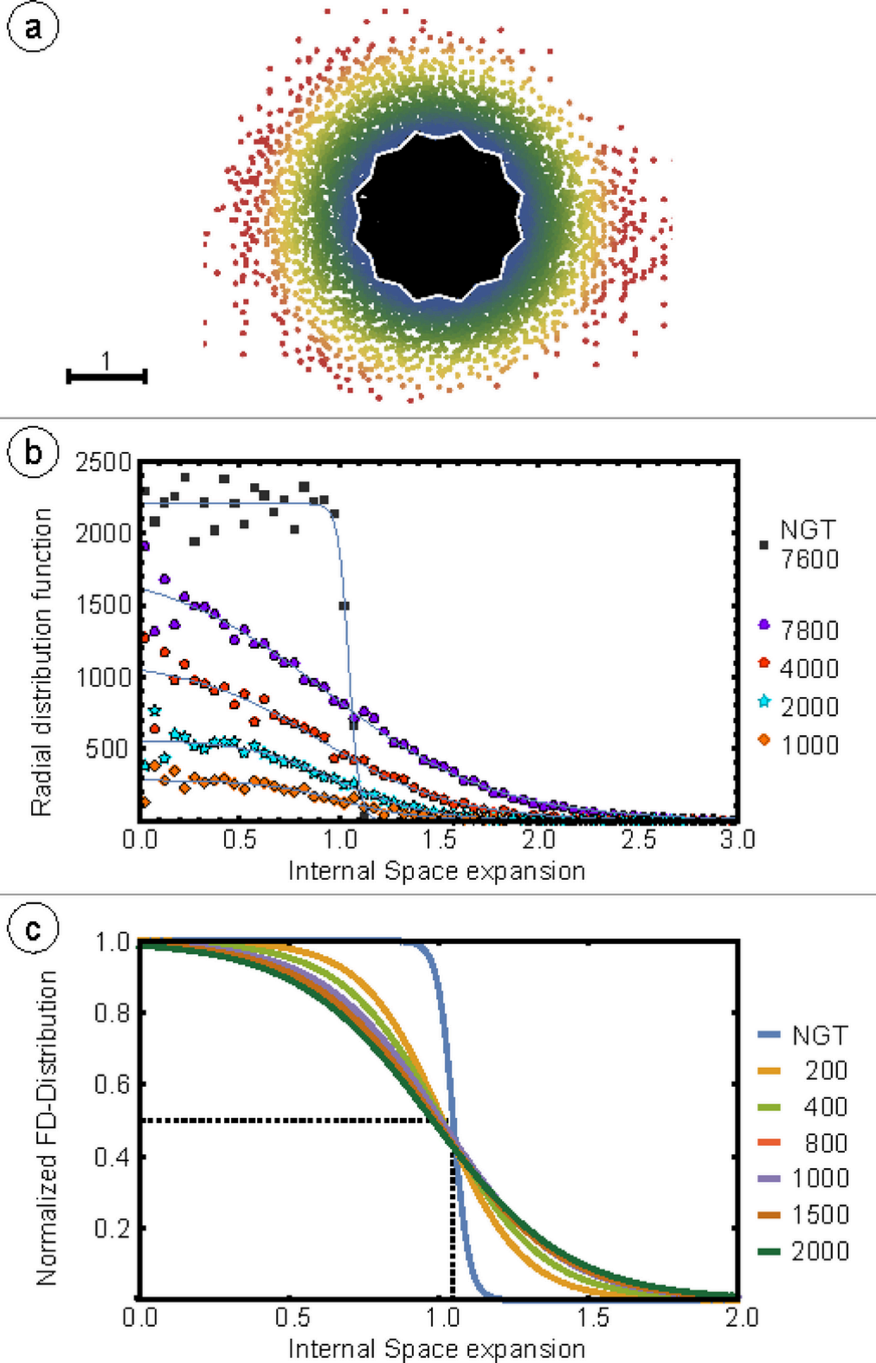
(*a*) 7800 vertices of the OQC in Ba–Ti–O/Pt(111) represented in internal space. The AD of the NGT is plotted in white in (*a*). The color gradient represents the internal space distance of points beyond the AD. (*b*) Evolution of the RDF in dependence of the number of vertices surrounding the center of the physical space image shown in Fig. 3 ▸. (*c*) Comparison of the normalized fits for different numbers of vertices.

Fig. 1 ▸(*a*) shows the point cloud of vertex positions in the 2D internal space, which is derived from a four-tuple indexing of the physical space vertices in Ba–Ti–O/Pt(111). The same physical space dataset was used previously for the statistical analysis of the physical space tiling (Schenk *et al.*, 2019 ▸).

All points within the AD are colored black in Fig. 1 ▸(*a*). Outside the AD, a color gradient ranging from blue to yellow to red indicates an increasing distance from the AD. Starting from 2.5 times the length of the projected unit vectors spanning the internal space, the vertices turn red. As Fig. 1 ▸(*a*) shows, the OQC is far from being an ideal quasicrystal. Despite the dense cloud of vertices in the center, additional vertices scatter homogeneously outside the AD. In addition, the density distribution in the internal space can be judged by its radial distribution function (RDF) for different numbers of vertices surrounding the central vertex in the physical space, as shown in Fig. 1 ▸(*b*). The data are derived from the area-weighted integration of the vertices in rings of constant width. For an ideal quasicrystal, one would expect a step-like function with constant vertex density up to the edge of the AD and zero density outside, as plotted for the NGT by black squares in Fig. 1 ▸(*b*). The experimental data show that the vertex density is highest at the origin of the internal space where the AD is centered. However, we observe a plateau of constant vertex density only for a total size of 1000 vertices (orange diamonds). For larger system sizes, the cloud of points smears out more rapidly around the AD boundary. These curves can be perfectly described by a Fermi–Dirac distribution (FDD). The solid lines in (*b*) are fits through the data. To facilitate comparison, Fig. 1 ▸(*c)* shows the normalized FDDs for smaller system sizes. The ideal NGT is described by an RDF that is symmetric about 1.05. It is not a step function because the AD is not a perfect circle. The star shape leads to a subtle broadening in the RDF. In all cases, the experimental data are symmetric to an internal space distance of 1.0. Increasing the size to 2000 vertices yields a constant decrease in relative vertex density between distances of 0.5 and 1 with respect to the AD center. Concurrently, an increasing vertex density appears at distances between 1 and 1.5 with respect to the AD center.

**Figure 2:**
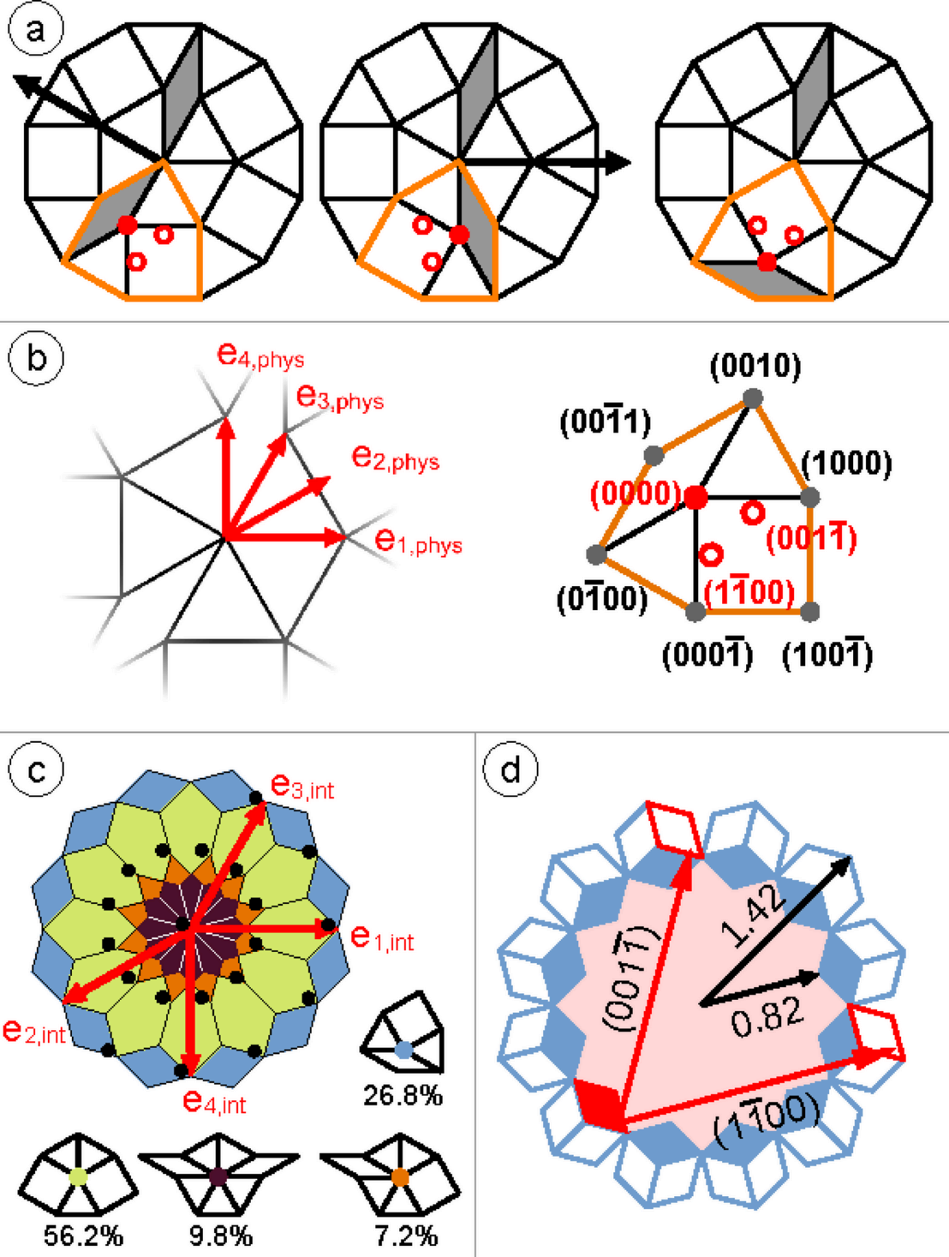
(*a*) Local triangle–square–rhombus tiling within the NGT. It consists of 20 vertices arranged in two rhombuses, five squares and 12 triangles. The orange-marked shield element encloses one rhombus, one square and two triangles with one vertex position inside the shield. A local mode or phason flip might move the inner vertex of the shield to the open red circle positions which does not change the tiling statistics. The sequence of three dodecagons illustrates such phason flips. (*b*) The unit vector definition in the physical space (left) and the corresponding four-tuple notation for the vertex positions in the shield (right). (*c*) The unit vectors defined in the internal space and the dodecagonal AD of the NGT. The latter is partitioned into colored areas depending on the local vertex configuration, as indicated just below. The blue area describes vertices within the shield that can undergo short-distance phason flips. The black dots represent 20 vertices of the characteristic dodecagon of (*a*). (*d*) The internal space representation of the area that hosts the vertices within the shield element (filled blue parallelograms) inside the AD and their potential flipped positions (empty parallelograms) outside the AD. The red arrows indicate the two possible phason jumps of the short-axis vertex labeled in (*b*).

The explanation for the scattering across the AD boundary is phason flips. Fig. 2 ▸ illustrates the simplest and most frequent phason flips that occur in the characteristic dodecagons of OQCs, as well as their consequences in the physical (*a*, *b*) and the internal space (*c*, *d*). These clusters consist of 20 vertices arranged into two rhombuses, five squares and 12 triangles. The long axes of the rhombuses form an angle of 150°. Because of this, a certain direction can be assigned to each dodecagon, as indicated by the black arrow in Fig. 2 ▸(*a*). Each vertex of the short axis of the rhombus is the inner vertex of a shield element, which is formed by two triangles, one rhombus and one square, as marked in orange in Fig. 2 ▸(*a*). This inner vertex of the shield (red full circle) can flip between three nearby positions without altering the tiling element statistics. The possible new positions are marked as red open circles in Fig. 2 ▸(*a*). As illustrated by the sequence of the three dodecagons, these phason flips correspond to 120° rotations of the shield element. Upon comparing the three clusters, it is evident that the central cluster is similar to the left one, but rotated by 150°. The right cluster differs because one rhombus moves to the periphery of the dodecagon when the shield is rotated. Fig. 2 ▸(*b*) provides the coordinate system spanned by the four unit vectors used for indexing the vertex positions. The four-tuple coordinates of all vertices within a shield element are given on the right side of Fig. 2 ▸(*b*). According to this definition, the short-distance phason flips inside the orange shield element correspond to moves along 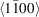 vectors, flipping the (0000) vertex either to (1100) or to (0011).

In Fig. 2 ▸(*c*) the AD of the ideal NGT is subdivided into differently colored areas corresponding to the four different vertex configurations, which exist in this tiling. As an example, in the green area in internal space all vertices are located that are surrounded by three triangles and two squares, also called 3^2^434 vertices. From the AD fraction of the green area the frequency of 56.2% is derived for these vertices. Please note that according to this subdivision 26.8% of all vertices are the ones forming the short axis of the rhombus and that those vertices are located at the AD periphery. The black dots in Fig. 2 ▸(*c*) mark the positions of the 20 vertices of the characteristic dodecagon. The cluster center is slightly shifted out of the AD center. The inner ring of 12 vertices in internal space corresponds to the outer vertices of the cluster in physical space. Focusing on the AD coloring one can see that two of these 12 are vertices surrounded by four triangles, two rhombuses and one square. The other ten are 3^2^434 vertices. The seven outer vertices in internal space form the inner ring in the physical space. Four out of these seven vertices are part of the short axis of the rhombus and three are 3^2^434 vertices that cannot perform the phason flip discussed in Fig. 2 ▸(*a*).

**Figure 3:**
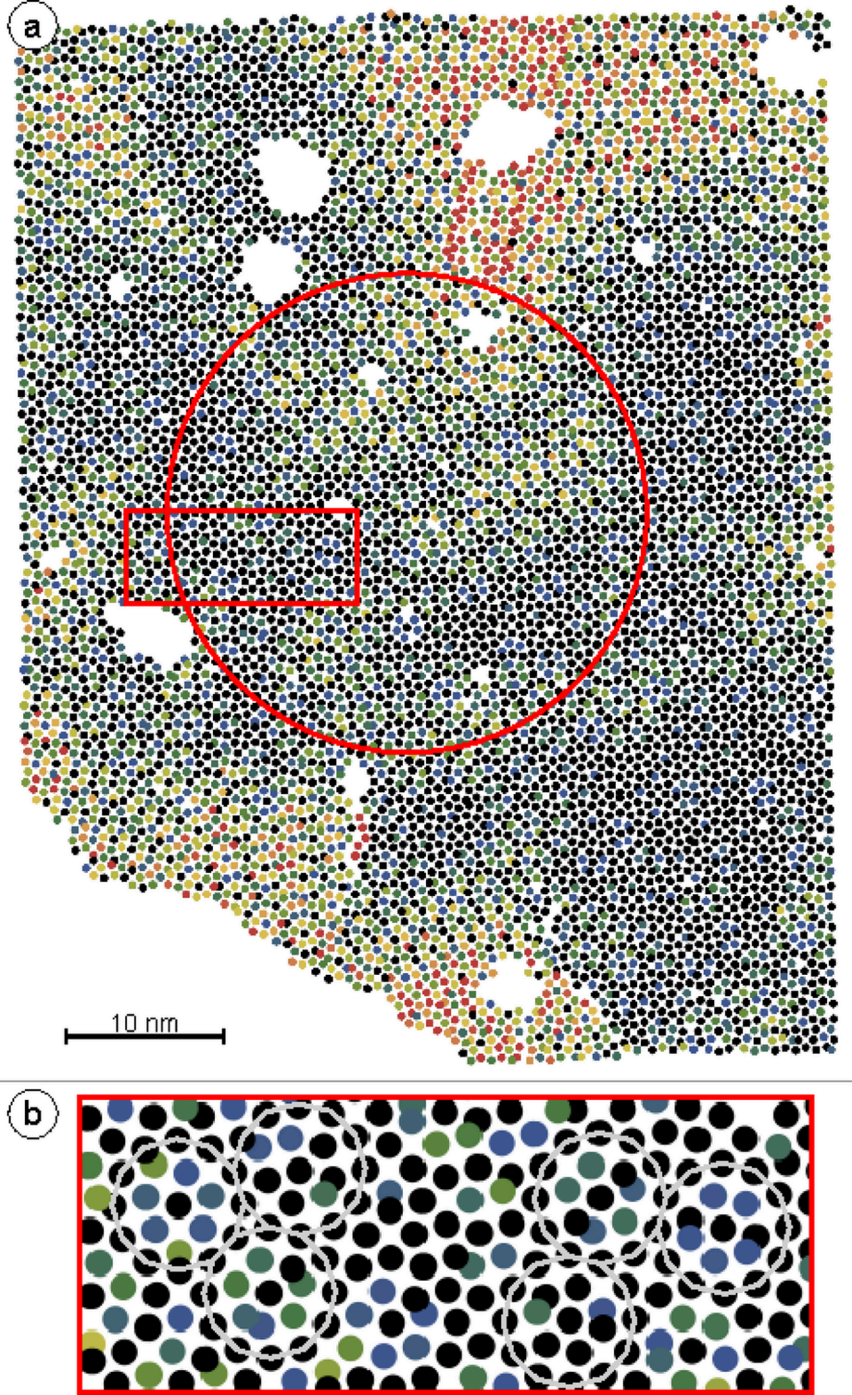
(*a*) 7800 vertex positions of the OQC in Ba–Ti–O/Pt(111) represented in physical space. The coloring of the vertices represents their internal space distance from the origin of the AD, as shown in Fig. 1 ▸(*a*). The red circle encloses 1800 vertices for which the internal space variance is presented in Fig. 4 ▸. (*b*) Enlarged view of the area inside the red rectangle in (*a*). Some characteristic dodecagons are marked as a guide to the eye.

Fig. 2 ▸(*d*) illustrates how the vertices are redistributed in the internal space upon short-distance 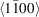 phason jumps. The filled parallelograms show the AD area fraction for the vertices of the short axes of the rhombuses. The empty parallelograms indicate the target regions outside the AD that are accessible for all possible rotations of rhombuses upon flipping. The two possible jumps discussed in (*a*) are marked in red in the internal space. These geometric considerations directly affect the RDF of the internal space. As the black arrows in (*c*) indicate, jumps of this type will cause a depletion of the vertex density in the AD above an internal space distance of 0.82 with respect to the AD origin, and an increase in density up to a value of 1.42. This trend is clearly evident in the experimentally derived RDF. Stronger evidence of the dominance of this mechanism in redistributing vertices in internal space comes from applying the internal space coloring to the physical space tiling, as shown in Fig. 3 ▸(*a*).

At first glance, the dark colors in the center of the physical space image reveal an ordered quasicrystal patch. Zooming into the region within the red rectangle of Fig. 3 ▸(*a*), as shown in Fig. 3 ▸(*b*), reveals that black vertices are often mixed with blue and green ones. This creates a central black dot surrounded by black, green and blue vertices inside a dodecagon made from 12 black vertices. This coloring depicts the physical space signature of the phason flips shown in Fig. 2 ▸. These occur frequently in OQCs and are a signature of local phason strain, as reported earlier in an extended statistical analysis (Schenk *et al.*, 2019 ▸).

More abrupt changes in the physical space coloring indicate a rapid movement over large distances in internal space. This may result from phason strain in an extended region or, in other words, from local inclusions of periodic approximant domain. Regions with red vertices appearing in the upper right and upper left parts of Fig. 3 ▸(*a*) illustrate this. Sudden jumps to red vertex colors in the central bottom or top parts are more likely to be related to topological defects, or dislocations, in the quasicrystal. These dislocations are associated with a Burgers vector, which defines an internal space length. Along this length, the vertex positions move away from the AD center (Lifshitz, 2011 ▸; Korkidi *et al.*, 2013 ▸).

**Figure 4:**
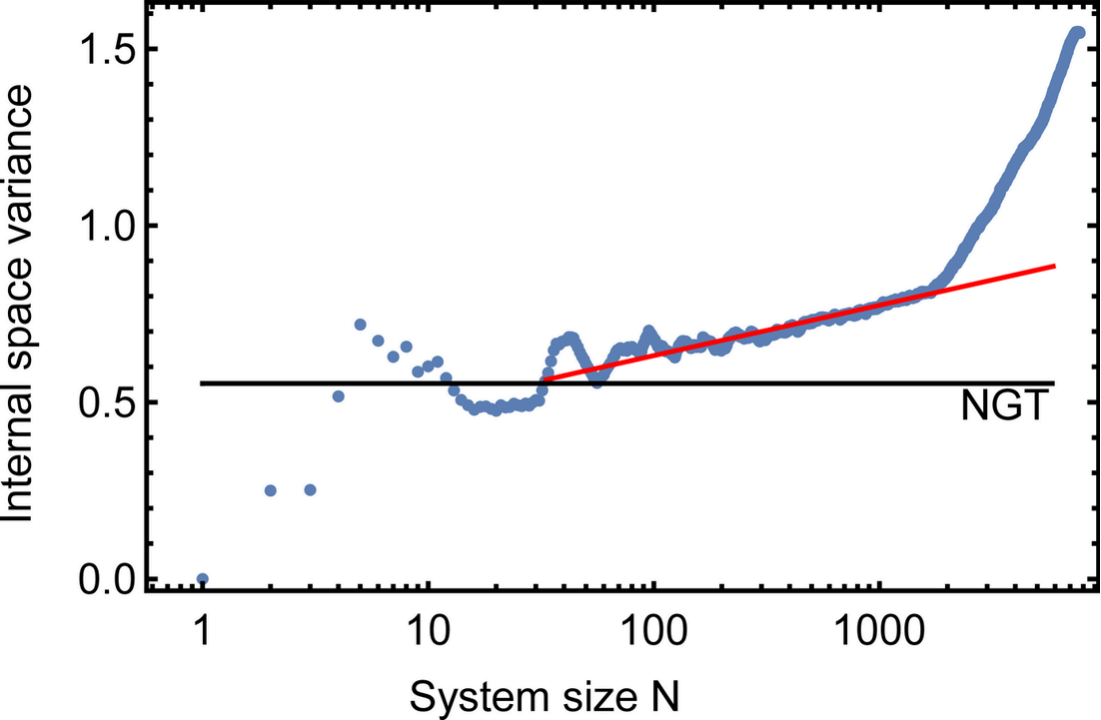
Variance of the internal space in dependence of the system size *N* for the OQC in Ba–Ti–O. The indexing started in the center of the red circle in Fig. 3 ▸(*a*). The black horizontal line represents the variance of the ideal NGT. The red line is a fit to the data between 10 and 1800 vertices.

The internal space variance 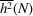 is shown as a function of system size *N* in Fig. 4 ▸. The black horizontal line indicates the constant variance of the ideal NGT, whereas the experimental data (blue circles) show a logarithmic increase up to a sample size of about 2000 vertices. Using equation (2), a logarithmic slope of 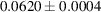 is obtained, which translates to an effective phason elastic constant of 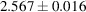. From a size of about 2000 vertices, the curve continues with a much larger slope. For the Ba–Ti–O dataset presented here, about 2000 vertices is the typical size of the well ordered quasicrystal patch. The red circle in Fig. 3 ▸(*a*) indicates the perimeter of this patch. The large number of yellow and red vertices beyond the red circle explains the change in slope with increasing system size. However, this evaluation depends only slightly on the starting point for indexing. Here the central vertex was used. Varying the starting vertex for this analysis, *e.g.* by picking the center of the extended dark area in the lower right of Fig. 3 ▸(*a*), yields a similar result.

The second OQC system to be discussed is Eu–Ti–O/Pd(111) (Schenk *et al.*, 2022 ▸). This system was fabricated by depositing Eu on a TiO_*x*_/Pd(111) precursor, which was prepared by molecular beam epitaxy of Ti and post-oxidation. The Eu–Ti–O/Pd(111) system exhibits a dodecagonal square–triangle–rhombus tiling similar to that of Ba–Ti–O/Pt(111). However, the average pore size of the Ti_2_O_3_ network hosting the Eu atoms is slightly smaller than that in Ba–Ti–O/Pt(111) (Schenk *et al.*, 2022 ▸).

**Figure 5:**
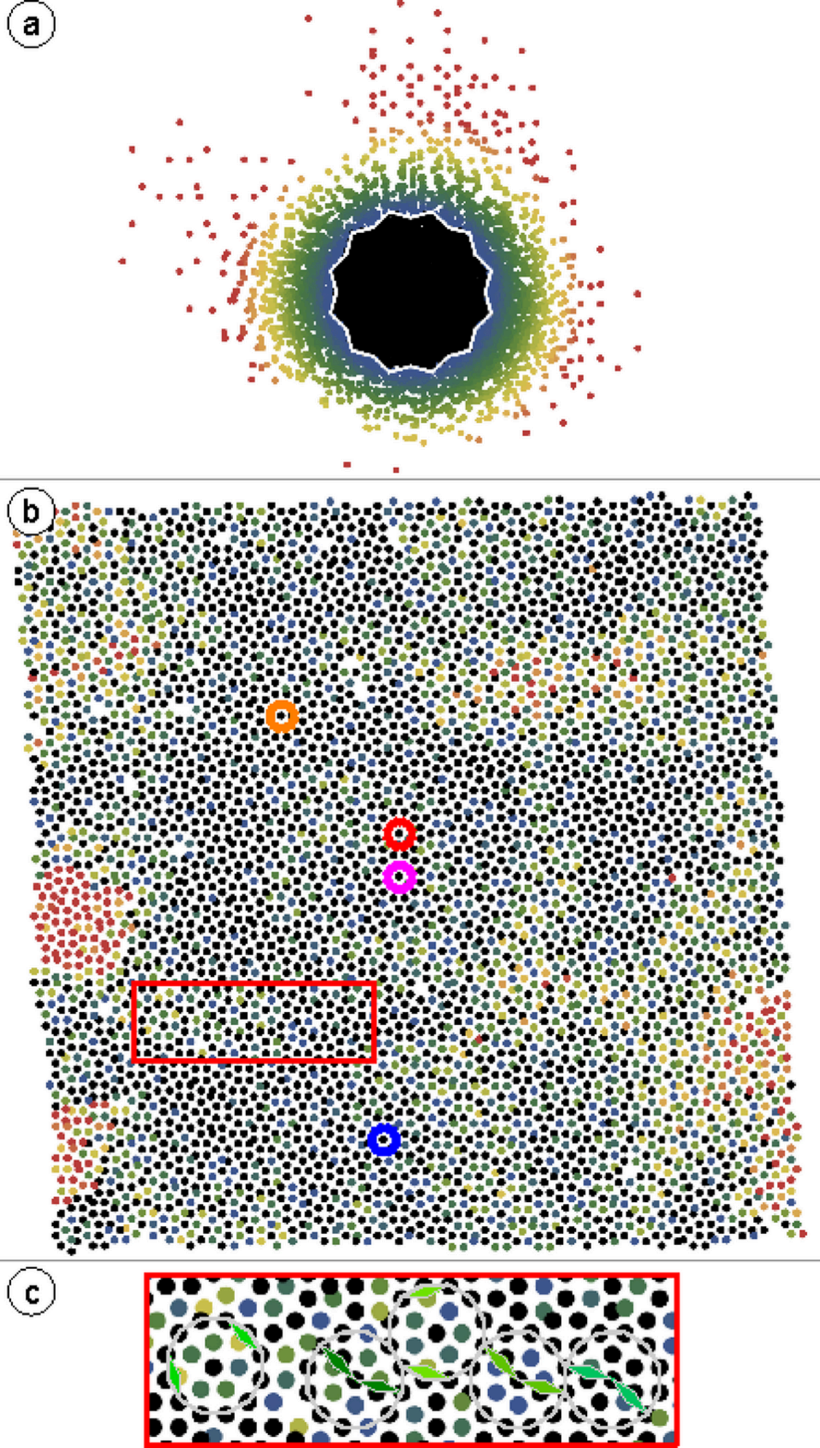
4800 vertex positions of the OQC in Eu–Ti–O/Pt(111) represented in internal space (*a*) and physical space (*b*). The border of the AD of the NGT is plotted in white in (*a*). The color gradient represents again the internal space distance of points outside the AD. The orange, red, pink and blue circles label the different central vertices for evaluating the internal space variance plotted in Fig. 6 ▸. (*c*) Enlarged view of the region inside the red rectangle in (*b*) emphasizing the position of rhombus pairs inside characteristic dodecagons.

Fig. 5 ▸ shows the internal and physical space of the OQC in Eu–Ti–O/Pt(111). This dataset contains 4800 vertices. Again the internal space vertex representation in Fig. 5 ▸(*a*) is a compact cloud with a certain fraction of vertex positions located outside the AD. The region directly around the AD, colored in blue and green, is densely populated, and the vertex density fades out towards yellow and red. Few red vertices are found at distances larger than twice the unit vector (radius of the AD) in three distinct directions. In physical space, Fig. 5 ▸(*b*), dark colors dominate a central strip from top to bottom. Towards the right, some colorful areas indicate larger phason strain. To the left, two red patches embedded in a high density of black vertices indicate a sudden jump in internal space. Upon closer inspection, rings of 12 black vertices surrounding a black center vertex indicate the characteristic dodecagon of the NGT and are present everywhere in the well ordered OQC patch. This is emphasized in the enlarged region of the red rectangle in Fig. 5 ▸(*c*). Unlike in Ba–Ti–O, phason flips result in configurations in which the rhombuses no longer point to the center of the dodecagon, but instead are flipped to positions on the periphery. However, these flips result in the same internal space scattering out of the AD, as discussed before [Fig. 2 ▸(*c*)].

**Figure 6:**
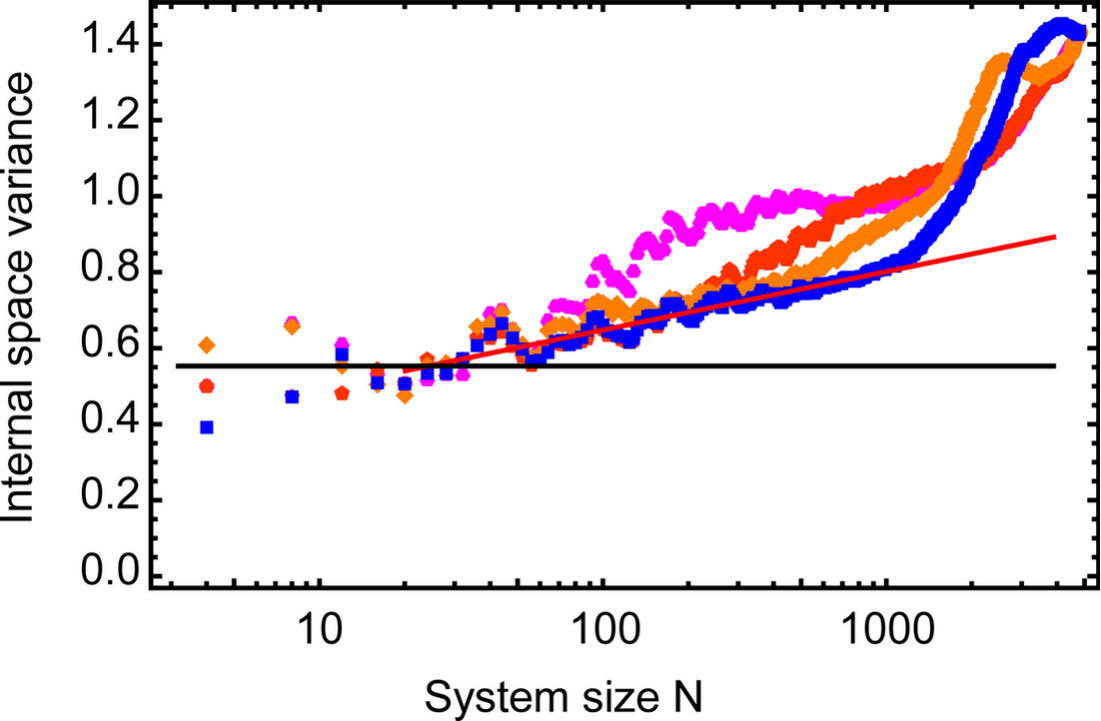
Variance of the internal space in dependence of the system size *N* for the OQC in Eu–Ti–O. The different traces are obtained by using different vertices as starting points for indexing. The starting vertices are labeled in Fig. 5 ▸ in the colors of the traces. The black horizontal line represents the variance of the ideal NGT. The red line is a fit to the data between 10 and 1000 vertices of the blue trace.

When evaluating the internal space variance for this dataset, we find that the system’s size dependence now strongly varies according to the starting vertex chosen. We illustrate this with four examples, indicated by colored circles in Fig. 5 ▸(*b*). The four curves showing the evolution of the variance with the system size are plotted in Fig. 6 ▸. The pink curve shows a rapid increase in variance that saturates at around 200 vertices, slowly decreasing to 1000 vertices before a significant increase. The red and orange curves show intermediate initial linear slopes, and the blue curve shows the slowest linear growth of variance in this system. By fitting the blue curve with equation (2) between 10 and 1000 vertices, we obtain a slope of 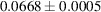. This translates to an effective phason elastic constant of 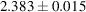, which is close to the value obtained for Ba–Ti–O.

**Figure 7:**
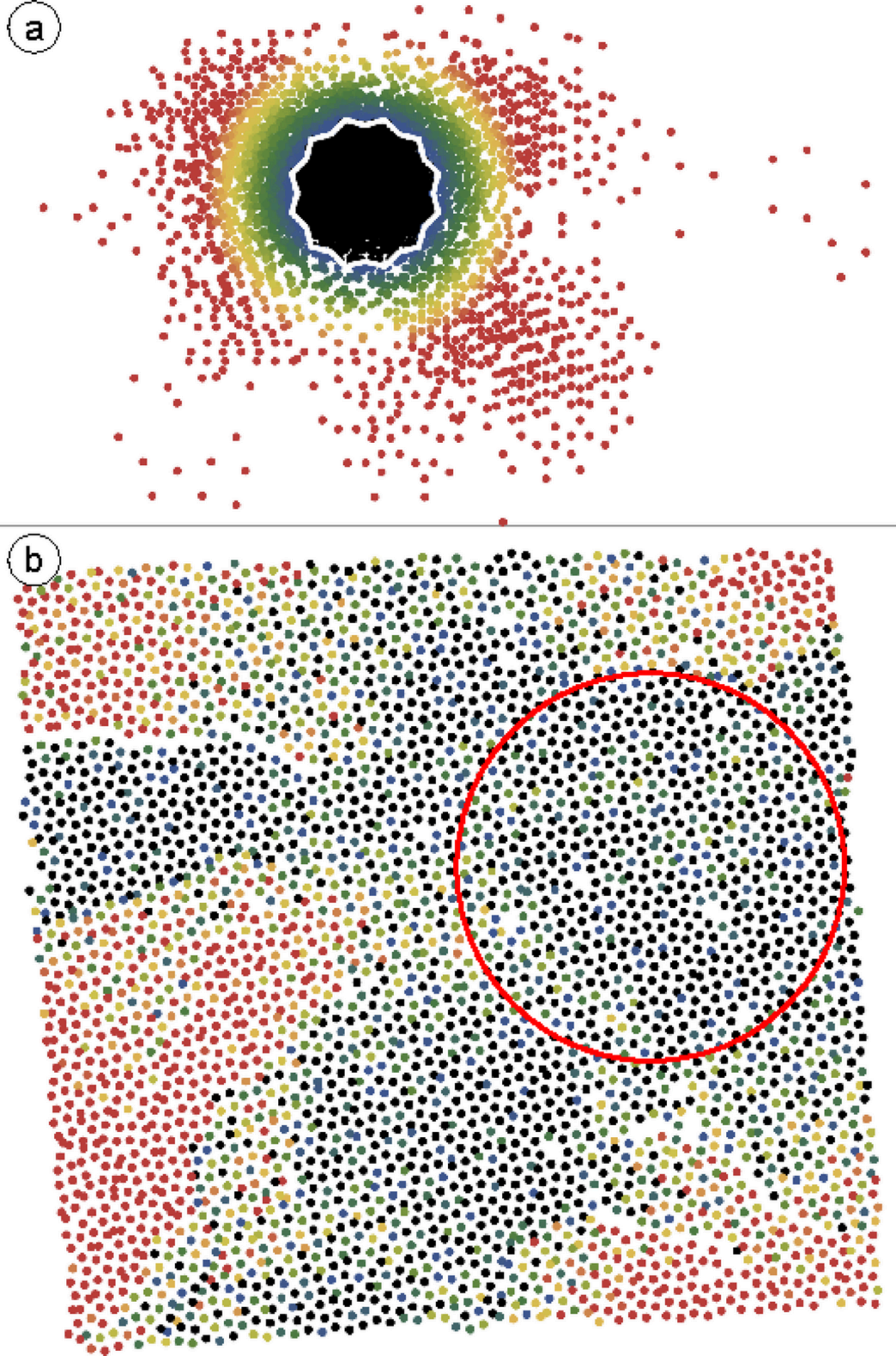
3600 vertex positions of the OQC in Sr–Ti–O/Pt(111) represented in internal space (*a*) and physical space (*b*). The AD of the NGT is plotted in white in (*a*). The color gradient represents the internal space distance of points beyond the AD. The red circle in (*a*) labels the largest well ordered continuous OQC patch containing 700 vertices.

**Figure 8:**
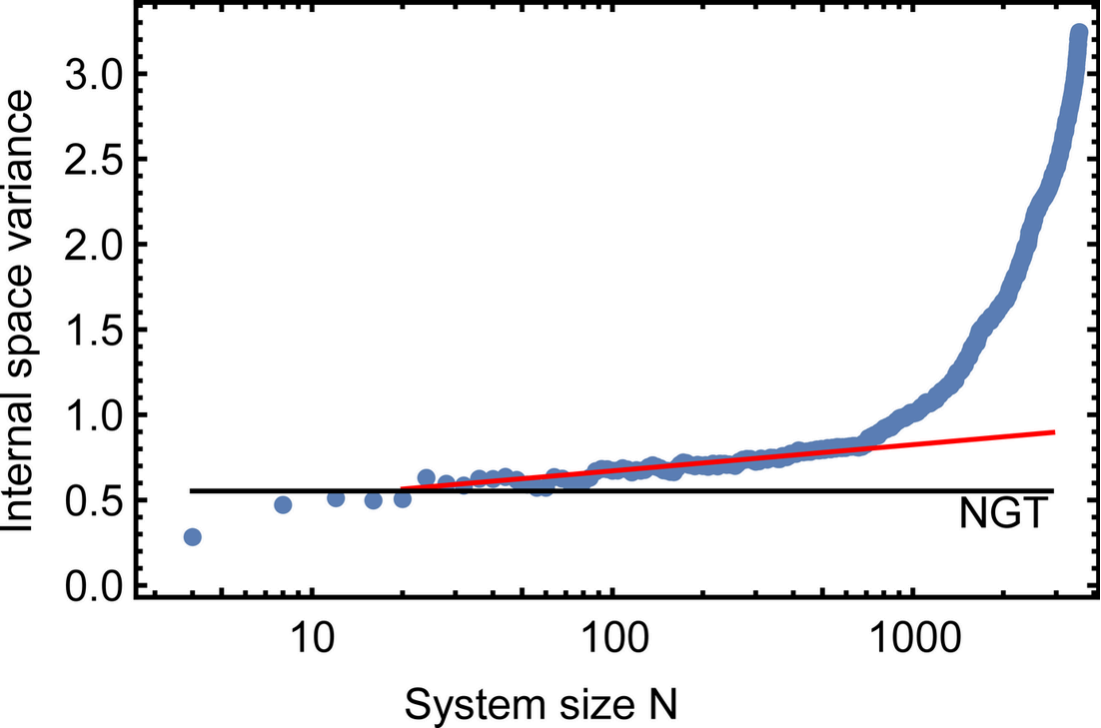
Variance of the internal space in dependence of the system size *N* for the OQC in Sr–Ti–O. The black horizontal line represents the constant variance of the ideal NGT. The red line is a fit to the data between 10 and 700 vertices.

The third system to be discussed is the OQC in Sr–Ti–O/Pd(111) (Haller *et al.*, 2025 ▸). Fig. 7 ▸ shows internal and physical space images of a dataset comprising 3600 vertex positions. The wide spread in the internal space indicates that this dataset shows less perfect quasicrystalline ordering. The regular distribution of red vertices surrounding the AD suggests that the presence of extended patches of quasicrystalline approximants is responsible for this. In physical space, Fig. 7 ▸(*b*), a strong separation into compact red and black regions is clearly visible. In the bottom left part of the image, the domain boundaries appear quite sharp due to a sudden jump in the vertex color. As was also the case in the other OQC systems, this jump corresponds to an extended shift vector in perpendicular space when crossing the domain boundary. The open line in the top left part of Fig. 7 ▸(*b*) shows that the indexing was not possible in this region, with gaps occurring in the tiling that cannot be bridged by the edge length of the prototiles. However, as indicated by the red circle, a continuous OQC patch of 700 vertices can be found in this dataset. The evaluation of the system-size dependent internal space variance shown in Fig. 8 ▸ started from the center of this circle. The curve bears a strong resemblance to the growth of the variance in the other two OQC systems. Up to 700 vertices, a logarithmic growth variance is obtained that can be fitted with a slope of 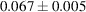, corresponding to an effective phason elastic constant of 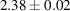. This value is similar to that of Eu–Ti–O. Beyond 700 vertices, a significant increase in the variance is obtained. As expected, given the wide spread in the internal space, the variance maximum for these 3600 vertices is much larger than for the previous two systems. It differs by a factor of two. In this regard, the Ba–Ti–O OQC dataset comprising 7800 vertices is the most compact in the internal space.

## 4. Discussion

The internal space inspection has allowed us to identify finite patches of long-range ordered quasicrystals in all three dodecagonal oxide systems: Ba–Ti–O, Eu–Ti–O and Sr–Ti–O. In all cases, a logarithmic increase in variance was found as a function of system size. Interestingly, the slopes of the Sr and Eu OQCs are identical, while the slope of the Ba OQC is lower. This result can be rationalized as follows. A larger slope indicates a higher degree of phason disorder (Niizeki *et al.*, 1994 ▸), expressed by phason flips in physical space (Edagawa *et al.*, 2000 ▸; Edagawa *et al.*, 2002 ▸). In the case of the OQCs, such reconfigurations are associated with the transformation of the underlying Ti–O ring network hosting Ba, Sr or Eu atoms (Schenk *et al.*, 2022 ▸; Cockayne *et al.*, 2016 ▸). The driving force behind this network reorganization is the electrostatic repulsion between neighboring host atoms. These atoms reside as doubly positively charged ions in the layer (Schenk *et al.*, 2022 ▸; Haller *et al.*, 2025 ▸). Therefore, the size of these ions, as represented by their ionic radii, may be crucial for determining the degree of phason disorder in the network. While the ionic radii of Eu and Sr are nearly identical (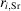 = 118 pm, 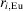 = 117 pm), the ionic radius of Ba (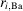 = 135 pm) is about 15% larger. The epitaxial relation to the substrate may also play a role. The lattice constant of Pd(111), as the template for Eu–Ti–O and Sr–Ti–O, is 0.8% smaller than that of Pt(111), the template for Ba–Ti–O. Consequently, the aperiodic Ti_2_O_3_ matrix hosting Eu, Sr or Ba ions expands from an average pore size of 6.70 Å on Pd(111) to ∼6.85 Å on Pt(111). These two factors should affect the difference in the effective phason elastic constants obtained for Eu–Ti–O and Sr–Ti–O with respect to Ba–Ti–O.

Values for the phason elastic constant 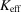 have been derived by modeling *e.g.* randomized Penrose-like tilings (Strandburg, 1989 ▸), a randomized Ammann–Beenker tiling (Joseph & Baake, 1996 ▸), or by using the transfer matrix approach (Widom *et al.*, 1989 ▸). These studies reported comparable values close to 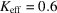. Experimentally, a value of 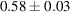 was derived for a molecular network described by a rhombus tiling (Blunt *et al.*, 2008 ▸). In all of these cases binary tilings were studied. For an intermetallic quasicrystal described by a Penrose-like tiling consisting of four prototiles, a larger value of 2.5 was found (He *et al.*, 1991 ▸). The small local phason flips of 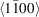 character allow excitations of the phason degrees of freedom with only a small expansion of the internal space variance. The latter results in a rather stiff effective elastic constant 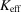. This is in contrast to larger rearrangements of the tiling, which are expected to occur in binary tilings.

**Figure 9:**
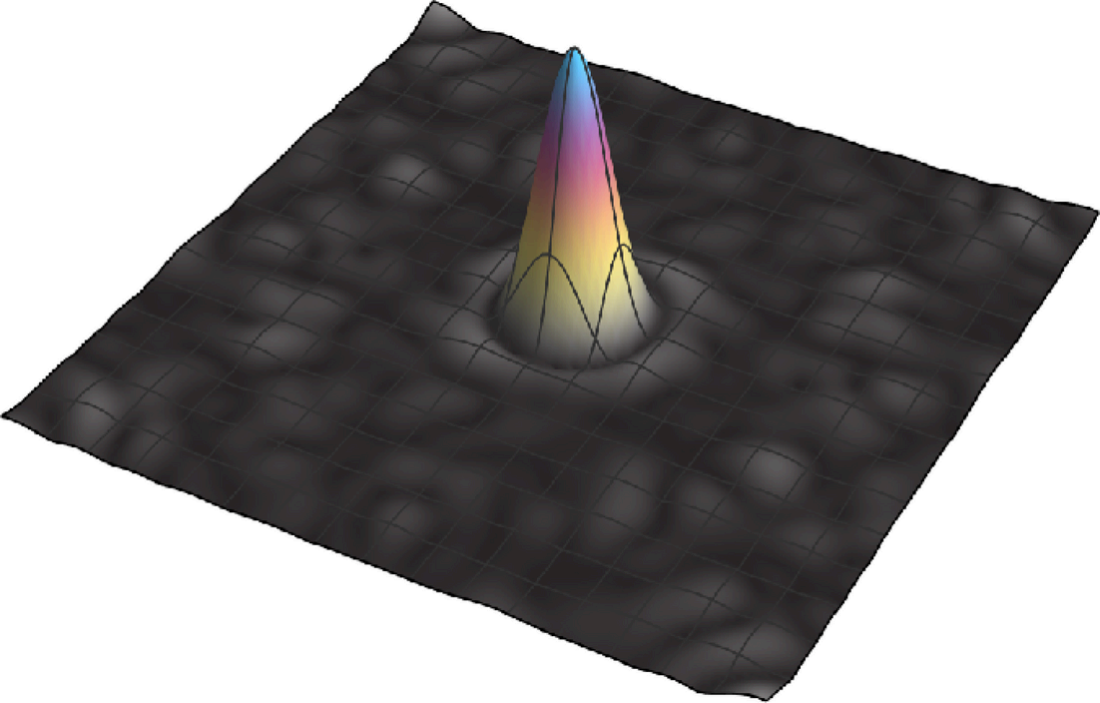
Absolute value of the discrete FT of the internal space distribution for the central 1800 vertices in Ba–Ti–O/Pt(111).

The logarithmic increase of the internal space variance might stem either from frozen-in phason waves or alternatively from a random tiling character. The detailed statistical analysis of the Ba–Ti–O system reveals rather ideal NGT properties when comparing especially the shared edges of neighboring tiles (Schenk *et al.*, 2019 ▸). This questions the random nature of the system and points to well defined local phason flips, which keep the number of shared edges unchanged. An additional tool for addressing this issue is the FT of the internal space distribution. The absolute value of the discrete FT of the internal space distribution for the OQC in Ba–Ti–O/Pt(111) is therefore shown in Fig. 9 ▸.

We observe a central Gaussian peak surrounded by a valley and a subsequent minor elevation, which indicates the onset of radial oscillations. Similar observations were made for the well ordered OQC patches in Sr and Eu OQCs. No oscillations are expected for a random tiling (Joseph & Baake, 1996 ▸; Joseph *et al.*, 1997 ▸). However, we also do not find coherent oscillations beyond this initial onset. Joseph *et al.* did not discuss this intermediate case, but other groups have reported it (Hory *et al.*, 1999 ▸; Soltmann & Beeli, 2001 ▸). These tilings were interpreted as being highly ordered, yet with some phason disorder. It is instructive to compare this situation with the convolution of the step function expected for the internal space vertex density for the ideal NGT with a Gaussian. According to the convolution theorem, the FT of the convolution is equivalent to the product of the single FTs, which are the oscillating function and the Gaussian. Consequently, the width of the Gaussian determines how quickly the oscillations are damped. A broader point cloud in internal space leads to a sharper Gaussian after Fourier transformation, resulting in stronger damping of the oscillations. In this case, the slope of 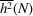 is relatively large, resulting in a comparatively small phason elastic constant 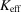. Conversely, if the slope of 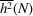 is small, then a large phason elastic constant follows. Consequently, the Gaussian in the Fourier-transformed internal space is broadened and the damping of the oscillation is weaker. Accordingly, the evolution of the RDF presented in Fig. 1 ▸(*b*) proves the narrow width of the broadening of the AD boundary in the Ba–Ti–O system, from which a weak damping of the oscillations in the FT follows. Thus, the increasing variance in the OQC systems can be traced back to the presence of phason disorder resulting predominantly from local 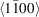 jumps of the vertex within the shields of the characteristic dodecagons.

## 5. Conclusion

We discuss the structure of dodecagonal oxide quasicrystals in the Ba–Ti–O/Pt(111), Sr–Ti–O/Pd(111) and Eu–Ti–O/Pd(111) material systems by uplifting the 2D physical space vertex positions as determined by STM into a 4D hyperspace. The internal space representation of the vertex positions reveals a homogeneous scattering of points beyond the acceptance domain, indicating the presence of phason disorder in these OQCs. The variance of the internal space increases logarithmically with the system size within compact OQC patches in all material systems. For the Sr–Ti–O and Eu–Ti–O OQCs, the logarithmically increasing variance translates to a common effective phason elastic constant of 2.38. For the Ba–Ti–O/Pt(111) OQC, a slightly larger value of 2.57 is obtained. This suggests that the OQC in the Ba–Ti–O/Pt(111) system more closely resembles the ideal square–triangle–rhombus tiling. Phason disorder originates from 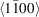 jumps of the vertex within the shields of the characteristic dodecagons in the tiling.

## Data Availability

The STM data presented in this article are available from the corresponding author upon reasonable request.
